# Visualization and Analysis of Mapping Knowledge Domains for Food Waste Studies

**DOI:** 10.3390/ijerph18105143

**Published:** 2021-05-12

**Authors:** Yiran Ouyang, Yanpeng Cai, Hongjiang Guo

**Affiliations:** 1 Guangdong Provincial Key Laboratory of Water Quality Improvement and Ecological Restoration for Watersheds, Institute of Environmental and Ecological Engineering, Guangdong University of Technology, Guangzhou 510006, China; Yiran.Ouyang@foxmail.com (Y.O.); guohongjiangzzl@163.com (H.G.); 2 Institute for Energy, Environment, and Sustainable Communities, University of Regina, Regina, SK S4S 0A2, Canada

**Keywords:** food waste, mapping knowledge domains, bibliometric analysis, evolution path

## Abstract

Food waste and loss is a global issue involving ethics, society, the environment, and the economy. However, there is a lack of systematic and visual scientific knowledge and graph methods to study the precedents of this field’s development over time. The article is based on the scientific knowledge graph research of articles published in the past 22 years to review the latest food waste research developments. The study will be conducted from the following perspectives: country/region, institution, author, journal, keyword co-occurrence, and article co-citation. It turns out that in the past eight years, food waste research has grown rapidly. A total of 8298 research articles have been published in 8064 journals and 176 Web of Science (WOS) subject categories. Research shows in the past 20 years. The main research hotspots were anaerobic digestion, biogas production, composting, biological hydrogen production, and innovation in system management methods. In the future, efficient and multitask biological value-added conversion technology, systematization of food-supply-chain decision-making aid models, and research on differences in management strategies may become the frontiers of research.

## 1. Introduction

Food waste(FW), mainly consisting of vegetable, offal, leftovers, and fruit husks is produced in our daily life from both household and industrial activities [1]. The United Nations Food and Agriculture Organization (UNFAO) reports that about 1.6 billion tons of food is lost or wasted globally every year, equivalent to one-third of all food resources produced by humans [2,3]. About 821 million people suffer from hunger; nearly 2 billion people do not have regular access to safe, nutritious, and adequate food. FW also implies the waste of relevant input resources in the whole food supply chain. [2]. The FW problem will not end here. Usually, FW treatment methods are landfill, incineration, and fermentation treatment. More than 95% of FW ends in landfills, where FW is converted into methane, carbon dioxide, and other greenhouse gases. Although many sites use landfill gas capture, its efficiency is only 68% [4]. The incineration of FW can prevent methane from being released into the atmosphere and recover heat to generate electricity. However, for some fruits and vegetables, the high moisture content of household food waste can lead to lower calories and even a negative lower heating value (LHV) [5]. Therefore, with in-depth research on FW, the severe economic and environmental consequences caused by improper handling of FW have attracted widespread attention from the international community. As an emerging research field, FW processing technology and management system have undergone tremendous changes. FW processing technology has transformed from a single task-oriented biological process to an integrated biological process with multiple tasks, including the simultaneous collection of resources, energy, and zero solid emissions [3]. The management perspective has changed from single terminal waste management to a whole-food-supply-chain system management [6]. The food management system itself has also been innovated, such as technological innovation [5,7], sustainable development [8], and life cycle assessment (LCA) [9]. At the same time, these increasing studies have been published in different journals in many subject categories. Moreover, some scientometric studies have been published to analyze the development trend of FW research [10,11,12,13]. This shows that food security is becoming a hot spot in environmental protection and resource research. Although the amount of academic literature continues to increase, there is a lack of methods to use more systematic scientific knowledge maps to study how this field has become a new research field over time. To solve this problem, this research adopts the scientific knowledge graph method, based on the quantitative analysis of peer-reviewed articles: (1) Summarizes the publications and scientific research cooperation of authors from major countries/regions and institutions in the FW research; (2) Analyzed the distribution of topics in FW research; (3) Determined the research focus and hotspot of FW research and defined the research trajectory over time; (4) Provided reference for the possible frontier challenges of FW research in the future.

## 2. Materials and Methods

### 2.1. Data Source

This article chooses the core collection as the data source for the study. The retrieval keywords were set “food-waste”, “food-garbage”, “kitchen-waste”, “kitchen-garbage”, “food-residue”, “kitchen-residue”, and the study period is set to 1998–2019. After excluding irrelevant documents (e.g., the related research of Archaeological Science, which is mainly about the food residues in historical sites), a total of 8298 papers were retrieved. These papers are organized by 5012 and published in 102 countries and regions; they span 176 Web of Science categories, contain 8064 publications and 21,641 authors. The types of documents Articles accounted for 81.8%, followed by Proceedings Paper (12.6%) and Review (7.5%). The number of other types of documents is almost negligible (see the Appendix A for the types of literature in FW studies in Table A1). Since papers can be divided into two different types, the total number used for statistics is greater than the number of articles retrieved. Throughout the research process, we mainly focused on the first three projects: articles, proceedings papers, and reviews.

### 2.2. Methods

Data were collected using the analysis function from the WOS core collection and were analyzed after drawing and tabulation. Moreover, knowledge domain maps for country co-authorship, organization co-authorship, author co-authorship, journal co-citation, and keyword co-occurrence were plotted using VOSviewer [14,15]. Reference co-citation knowledge domain maps and keyword timelines were plotted using CiteSpace [16].

## 3. Results

### 3.1. Yearly Quantitative Distribution of the Literature

**Figure 1:**
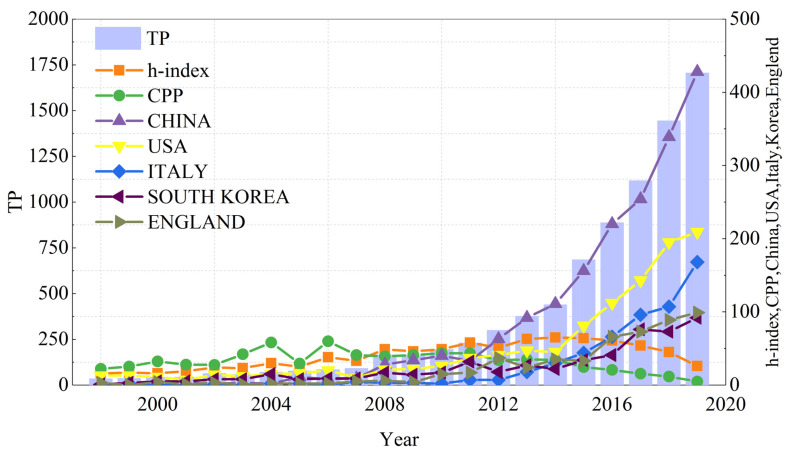
Annual variation curves for total annual publications (TP), h-index, citations per paper (CPP), China, USA, Italy, South Korea, England.

Changes in its literature output can measure the development status, knowledge accumulation, and maturity of a research field. The time-series output distribution of global FW-related literature is shown in Figure 1. In general, the research field of FW shows an overall upward trend, which can be roughly divided into three stages: (1) initial stage (1998–2007) in which total annual publications (TP) fluctuate slowly, and an average of 59.7 articles is published each year; (2) consolidation stage (2008–2014) in which the average number of articles published is 266; (3) rapid development stage (2015–2019) in which the number of papers increased from 685 in 2015 to 1705 in 2019, nearly 1.5 times. From the upward trend, the problem of FW has been noticed by researchers.

The annual h-index and citations per paper (CPP) are also shown in Figure 1; both indicators are calculated from the *Citation Report* of WOS. The curves for the h-index and CPP generally show a fluctuating trend. The h-index reached its maximum value in 2014, with a value of 65, meaning that at least 65 articles published in 2014 were cited at least 65 times. The maximum CPP was 59.81 in 2006.

The annual publications of the top five countries are also shown in Figure 1. It can be seen that China’s growth trend is highly correlated with the global FW publication trend, indicating that the volume of China’s FW publications has contributed a lot to the growth of the world’s FW publications. The concern of Chinese researchers in the FW field may be related to the policies and initiatives of the Chinese government. China has put forward the initiatives called “opinions on food conservation and anti-food waste (2014)” and then promulgated the “anti-food waste law (2020)”. Similarly, the United States of America (USA) and Italy’s growth trends may be due to increased national interest in improving FW management. In 2015, the U.S. Department of Agriculture (USDA) and the U.S. Environmental Protection Agency (EPA) announced the first goal of reducing food waste. The plan requires the United States to reduce food waste by 50% by 2030, and then the US government proposed an initiative in 2018 called “*Winning on Reducing Food Waste Initiative*”. The Italian government announced law no. 166/2016. Which purpose “to reduce waste for each stage of food production and supply.” All of these initiatives show the efforts of the national level on “anti-food waste”.

### 3.2. Quantitative Analysis of Production Countries/Regions

**Figure 2:**
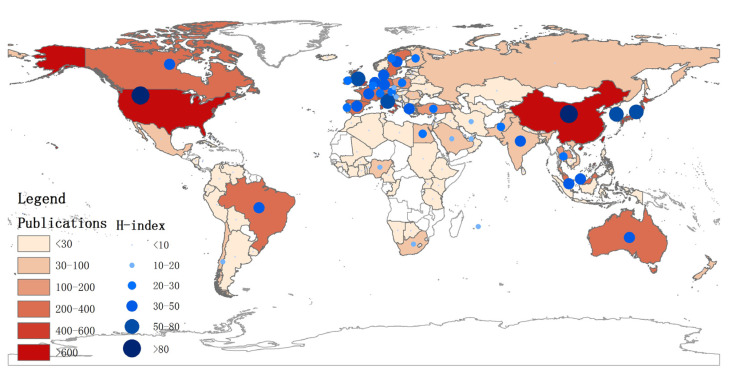
Country/region distribution of the literature.

**Figure 3:**
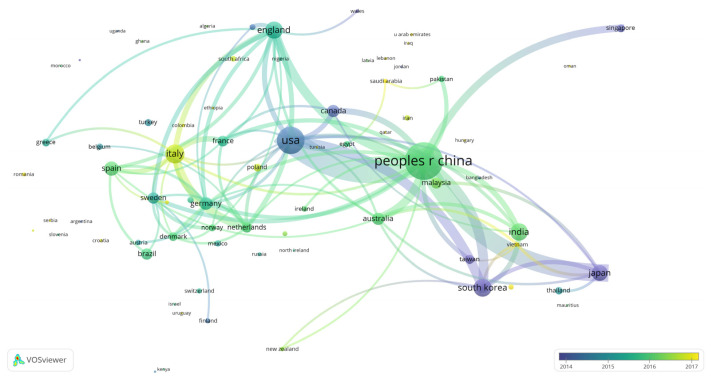
Mapping knowledge domains of coauthoring countries/regions in FW studies.

To clarify the main countries/regions participating in FW research and their cooperation, the distribution of countries/regions is analyzed. The country/region distribution map and the cooperative country/region knowledge map are shown in Figure 2 and Figure 3, respectively. As many as 102 countries in the world have participated in research on food waste. There are many FW studies in East Asia, South Asia, Eastern Europe, North and South America (Brazil). There are relatively few studies in Africa and South America. Countries with better economic conditions can give more support and attention to the scientific research of FW. China has the most documentary works, followed by the USA, Italy, and South Korea. These countries published 1843, 1110, 564, and 533 publications. Although China has the most articles, CPP is slightly lower than that of the USA, South Korea, and the United Kingdom. The most cited is the United Kingdom, followed by the USA and South Korea. The h-index indicator can indicate the influence of the country’s FW-related articles. It can be seen from Figure 2 that influence of FW in European and American countries is generally bigger. China, Japan, and South Korea have a bigger influence in Asia. It is worth mentioning that, as a populous country, India’s efforts in the field of FW research are worthy of recognition.

The h-index value is based on a list of publications ranked in descending order by the times cited count, which can quantify the author’s contribution to developing the FW field. It can be seen from Figure 2 that European and American countries have made more remarkable contributions to developing FW. In contrast, Asian regions, China, Japan, and South Korea, have made more significant contributions to developing FW. It is worth mentioning that India’s efforts in FW research are worthy of recognition as a country with a large population.

The map of knowledge domains for collaborative country/region creation is shown in Figure 3. The node represents different countries/regions, and the node’s size represents the activity intensity and the number of articles. The link between the two nodes indicates that there is a cooperative relationship between them. The shade of the color means the average release time. The closer the ties between the two countries/regions are, the stronger the cooperation will be. As shown in Figure 3, in terms of cooperation regions, intracontinental collaboration mainly highlights two groups: European and American cooperation groups (Italy–Netherlands, Italy–England) and Asian cooperation groups (China–Japan, China–Korea). InterContinental cooperation is primarily reflected in the association between China and the United States. In terms of period, the research on FW in the United States, South Korea, and Japan started earlier, while the average time of study on FW in China and Italy is after 2015, but related research is faster.

### 3.3. Research Institutions Focused on Quantitative Analysis

**Figure 4:**
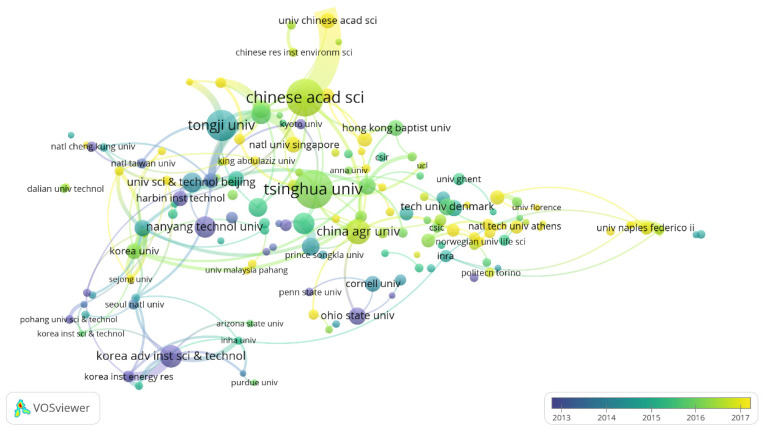
Mapping knowledge domains of coauthoring organization in FW studies.

Through the analysis of organization cooperation, we can determine the most productive research organization information for a specific topic [17]. To discover the leading research organizations, we listed the top 10 organizations that published the most papers (Table 1) and used VOSviewer to draw a knowledge domain map of collaborative organizations (Figure 4). As shown in Table 1, the Chinese Academy of Sciences (CAS) has the most significant number of articles, followed by Tsinghua University and the Tongji University. Tsinghua University, Tongji University, and the French National Institute of Agriculture, Food and Environment (INRAE) are all high in the Publications (Ps), h-index (H), and CPP rankings, indicating that their articles have a high volume of articles and a high average level. It is worth mentioning that the Korea Institute of Science and Technology (KAIST) and the University of California system (CU) are ranked eighth and seventh, respectively, in the Ps ranking. Still, they are in the top two positions in the CPP ranking. This shows that the average publication quality of these two organizations is relatively high. One more thing to note is that CAS shows low CPP in research institutions, indicating that some related articles published by CAS have quality problems. As shown in Figure 4, the results show that the nodes of KAIST, The Ohio State University, Nanyang Technological University, and Harbin Engineering University are dark blue, indicating that these universities’ research started early.

### 3.4. Authors of Hot Literature

**Figure 5:**
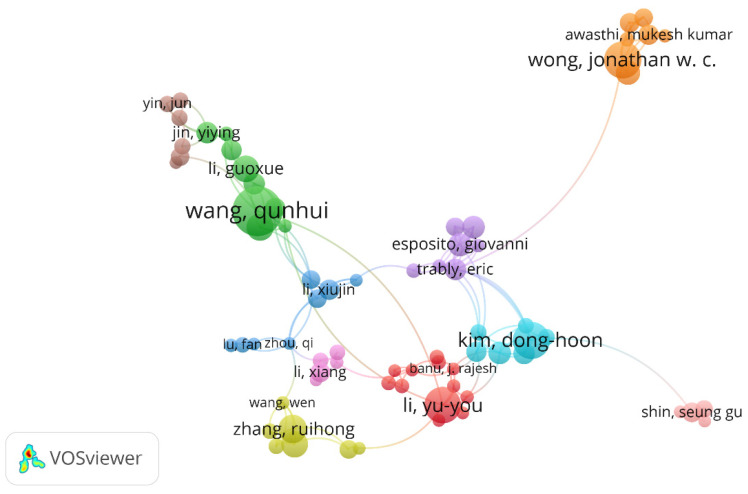
Knowledge domain map for the co-authorship network in FW studies.

Creating and analyzing knowledge maps for the co-authorship network can help research organizations develop cooperative groups. A knowledge domain map for co-authorship was plotted using the VOSviewer software, as shown in Figure 5. Each node represents an author, and the node size represents the number of published articles (see the Appendix A for the top 10 authors with the most publications in FW studies in Table A2). The link between two nodes indicates collaboration, and more connecting lines indicate closer collaboration between authors [14]. The author cooperation map in the FW field reflects the following characteristics: (1) The author cooperation in the FW field is mainly domestic; Wang Qunhui (University of Science and Technology Beijing), Li Xiujin (Beijing University of Chemical Technology), Li Guoxue (China Agricultural University), Jin Yiying (Tsinghua University) and other Chinese scholars cooperate closely. (2) As far as the prominent authors of FW research in China are concerned, Wong Jonathan W.C. (Hong Kong Invasion University), Zhang Ruihong (Harbin Institute of Technology), and other major domestic research institutions have little cooperation. (3) There is still a small-scale international collaboration, and the principal international cooperation authors include Kim Donghoon (Inha University, South Korea), Esposito Giovanni (Parma University, Italy), and Li Yuyou (Tohoku University, Japan).

### 3.5. Quantitative Analysis of Major Source Journals and Subject

**Figure 6:**
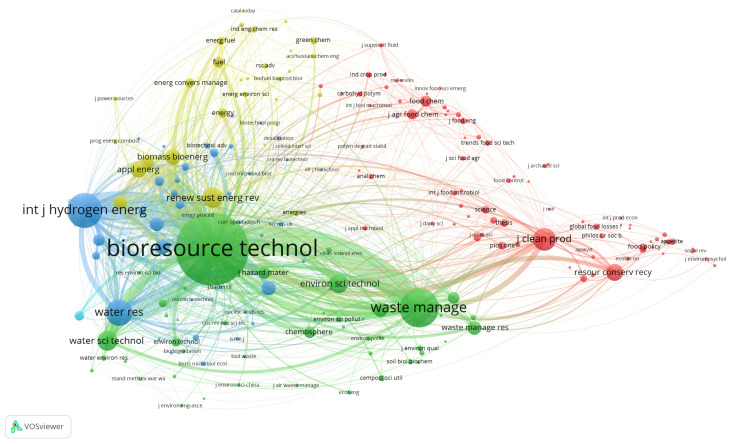
Mapped knowledge domains for journal co-citation in spontaneous combustion studies.

Journals are an essential source of academic exchanges and the dissemination of scientific achievements. By analyzing the journals’ distribution, we can determine the core journals in a certain field and the main research topics in this field. Co-citation of journals is described by mapping the knowledge domain of co-citation journals, as shown in Figure 6. The size of the node indicates the number of articles published by the journal. The line connecting the two journals indicates that two journals are cited in the same paper, and the thickness of the line represents the citation strength of the two journals. The largest node is *Bioresource Technology* (B&T), followed by *Waste Management* (WM), *Journal of Clean Production* (JCP), and *International Hydrogen Energy Magazine* (IJHN). Although IJHN has fewer publications than the first three journals, IJHN’s CPP ranks first (see the Appendix A for the journals with the most publications in FW studies in Table A3), which shows that the direction of biological hydrogen production has significant potential in the field of FW. In terms of co-citation strength, the connection line between B&T and IJHN is the thickest, followed by B&T and W&M. The possible reason for this correlation may be because FW management has become an effective method to achieve the dual goals of waste reduction and energy production [18]. The themes of these journals are mainly biotechnology, environment, energy, and sustainable development. These themes have an essential impact on FW research (see the Appendix A for the top 10 disciplines with most publications in FW studies in Table A4).

### 3.6. Keyword Co-Occurrence Analysis

**Figure 7:**
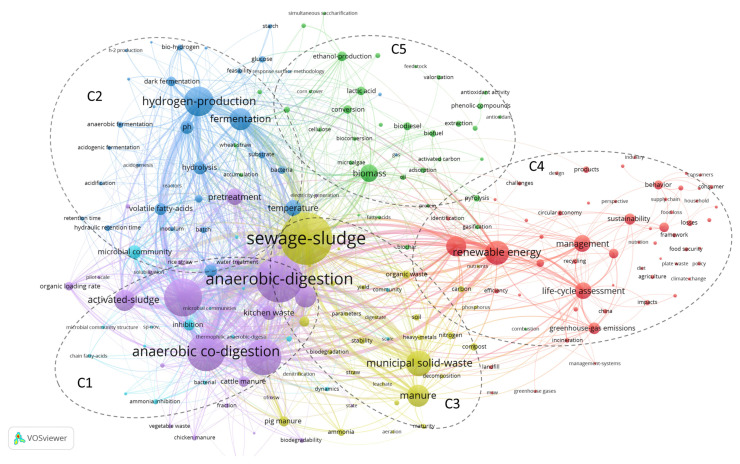
Knowledge domain map of a keyword co-occurrence network in FW studies.

Keyword co-occurrence analysis is used to describe the core content and structure of a certain academic field and reveal the subject’s research frontier. The keywords used for FW research are extracted from the core database of Web of Science. The maximum size of the database is 22,749. Due to the existence of many initial keyword data and synonyms, use “t thesaurus_terms.txt” to delete synonyms and set the minimum simultaneous occurrence threshold to 43 to obtain 191 keywords. As shown in Figure 7, the keyword co-occurrence analysis highlights five main clusters, each representing different research directions of FW. The critical node size represents the frequency of the key. The thickness of node connections indicates the strength of co-occurrence between keywords.

- Cluster 1 (C1) includes “anaerobic-digestion”, “anaerobic co-digestion”, and “biochemical methane potential”, which represents the most common research field in FW. “Anaerobic digestion (AD)” refers to the rapid degradation of complex organic matter into biogas through anaerobic microorganisms’ metabolic activities under hypoxia conditions [19]. “Anaerobic co-digestion” refers to the process of co-digesting FW with other organic feeds (such as animal manure, crop straw, and activated sludge) to improve AD efficiency and reduce reaction residues [20].
- Cluster 2 (C2): The core keyword is “hydrogen production”, its total connection strength is 5166, and the number of co-occurrences is 913. Food waste is rich in carbohydrates and protein, with a balanced nutrient ratio. It is an ideal substrate for anaerobic hydrogen production [21]. Hydrogen production by anaerobic fermentation is a new type of hydrogen production method that uses anaerobic heterotrophic bacteria and organic matter as a substrate to produce hydrogen. Its mild reaction, high hydrogen production capacity, and strong applicability of raw materials have become a resource for kitchen waste [22].
- Cluster 3 (C3) is mainly related to the field of aerobic composting, and its keywords include” manure”, “pig manure”, and “compost”. The essence of aerobic composting is a dynamic process of various microorganisms working together under aerobic conditions to convert difficult-to-decompose organic matter into stable humus. FW usually contains a high concentration of easily degradable organic substances, such as sugar, starch, lipid, and protein, and is a high-quality composting material [23].
- Cluster 4 (C4): It can be seen that C4 mainly represents macrolevel management methods, including “model” and “life-cycle assessment”. “Life-cycle assessment” has been proven to play an important role in research on the environmental impact of food waste and sustainability [9]. Research on consumer behavior in the food supply chain has also begun to emerge. The level of social education and publicity management often determines the amount of food waste to a certain extent [24].
- Cluster 5 (C5) mainly focuses on the keyword “biomass”, followed by keywords such as “ethanol production”, “lactic acid”, “biodiesel”, and “phenolic compounds”. This clustering mainly reflects another direction in the field of food waste research is the biorefinery of other high value-added chemicals, including activated carbon adsorbent, antioxidants, bioactive, fuels, biomaterials, corrosion inhibitors, enzymes [6,25,26,27,28]. It can be seen that compared with other clusters, there is less research in this direction, which is mainly due to the high economic cost and low output efficiency.

## 4. Evolution Path Analysis

### 4.1. Reference Co-Citation

**Figure 8:**
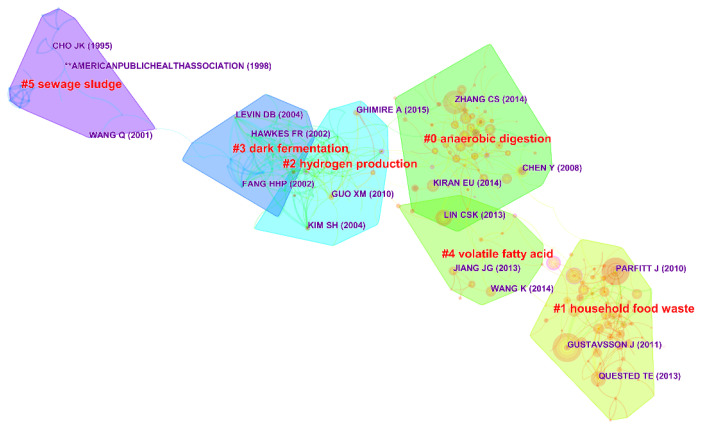
Knowledge domains map for reference co-citation.

The concept of co-citation was proposed by the American intelligence scientist Henry Small [29]. Document co-citation means that an article is cited by two articles at the same time. The knowledge base of the research field can be determined by co-citing documents [30]. We used CiteSpace software to draw a co-citation knowledge network graph and generated clusters that change over time, as shown in Figure 8. The darker the color filled in the cluster, the earlier the research topic will be formed. The co-citation network of documents is intricate, and highly overlapping parts and scattered parts constitute research branches. The nodes in Figure 8 represent different cited documents, and the larger the node, the more cited documents. Figure 8 shows a total of 5 main clusters.

Cluster tags are typical keywords obtained through different algorithms in the field of food waste. The numbers in parentheses indicate the average age of the cited references. The earliest research is the “sewage sludge” (#5, 1999). The research of cluster 5 mainly includes the treatment technology of various food wastes and how to convert the treated residues into fertilizers for reuse. Subsequent themes were dark fermentation technology (#3, 2002) and biological hydrogen production technology (#2, 2007). Hydrogen production by fermentation refers to a process in which organic waste is converted into organic acid by anaerobic heteroerobes under anaerobic conditions for methane fermentation, and hydrogen is obtained as a byproduct [31]. This also means that researchers have begun to explore more possibilities for recycling and reuse of food waste from the perspective of new energy sources for FW hydrogen production. In addition, research on reducing environmental pollution emissions and obtaining the required final products at operating costs (as cheaply as possible) also has attracted more attention from the government and related researchers. Similarly, anaerobic digestion (AD) (#0, 2011) proved to be an effective solution for FW treatment and value-added [4]. Compared with other traditional FW treatment methods, AD has a better treatment effect due to its limited environmental footprint, high energy-recovery potential, and organic fertilizer or biological fertilizer carrier materials [32]. To further increase the biogas production, accelerate the degradation rate and reduce the amount of final residue, many work has been done to help understand the most suitable fermentation conditions, such as pH, temperature, volatile fatty acids (#4, 2012), and pretreatment [33,34]. The latest cluster is household food waste (#1, 2013), which mainly includes macrolevel assessment methods (life-cycle assessment, waste hierarchy, carbon emission assessment), management methods (waste management, sustainability, policy framework), etc.

### 4.2. Research Front Identification

**Figure 9:**
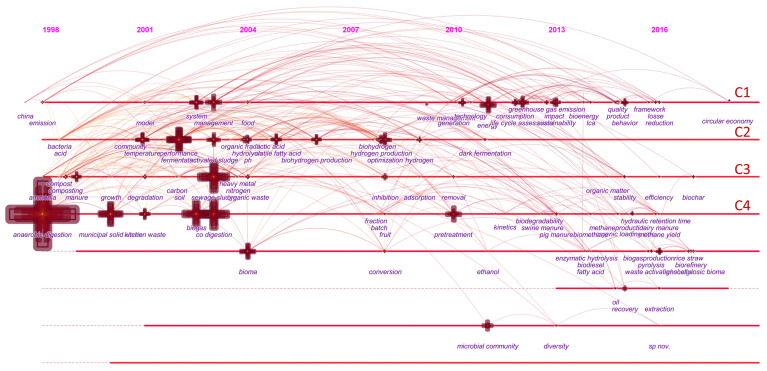
Keyword timeline view of food waste studies.

The timeline view shows the clusters of each research progress and can show developing research topics. The Y-axis is the keyword cluster, the X-axis is the release year, and the horizontal lines of the image parallel to the X-axis represent different key clusters [35]. By detecting burst keywords, a timeline view can be generated, as shown in Figure 9. Burst keywords mean that the keyword is frequently used in a short time, and the visual performance is the darker the color of the node, the stronger its burst. The link indicates that there is a connection between the two nodes.

According to the time-series analysis, the main features are as follows: Cluster 4 (C4): “anaerobic digestion (1998)” appeared and was widely used in the field of FW. After this, “biogas” appeared in 2002 as the main product of FW resource reuse. The improved process of anaerobic digestion, “co-digestion” technology, and “pretreatment” technology have played a role in reducing reaction time, reducing reaction residues, and improving reaction efficiency. Important role [34,36,37]. Subsequent research also focused on optimizing AD reaction parameters (hydraulic retention time), related physical and chemical properties (biodegradability), and their purpose is to make the biogas production process more stable and efficient [33,38]. At the same time, it also shows that the anaerobic digestion biogas engineering technology can achieve the goals of developing a circular economy, environmental protection, and producing renewable energy. Therefore, as the beginning of research in the FW field, most researchers studied the process of food waste treatment from the perspective of anaerobic digestion. In the future, in FW anaerobic digestion, more targeted, efficient parameter optimization and lower reaction cost will still be the important research direction of FW. At the same time, it is worth noting that the current resource utilization is mostly the product of a single technology and a single target, which may underestimate the resource potential of FW, and the coupling of multiple technologies and the output of the whole processing chain will give more possibilities. Cluster 3 (C3): In 1999, there were three synonymous keywords, “manure/composting/compost”, which indicated that the research on food waste in fertilizer production started in 1999. This is also the initial field of FW resource recycling, even earlier than the emergence of “biogas”. Although composting is not a new waste treatment method, the basic knowledge of FW composting is still relatively lacking, so the characteristics of FW still pose unique challenges for researchers [23,39]. It is worth noting that after 2007, no new research hotspots appeared in this field, and related research gradually decreased. In the future, the development of FW composting should not be limited to the research of reaction mechanism, but the system-wide control from “garbage sorting and recycling” to “fertilizer quality “. Cluster 2 (C2): Compared with clusters 3 and 4, whose main findings were concentrated before 2004, the research on “biohydrogen” before 2010 has been more active, and new research hotspots are constantly being proposed. Cluster 2 is mainly studies of the optimal operating conditions of biological hydrogen production, such as temperature [40], microbial community, pH [41], volatile fatty acids, hydrolysis, hydraulic retention time [37]. This shows that the use of FW to produce hydrogen through biological pathways has become an important field of FW research. As a sustainable energy source, hydrogen is an effective substitute for fossil fuels. It is a clean and environmentally friendly fuel that produces water instead of air pollutants (NOx, CO) when burned. With the increase in energy demand and global attention to global climate change, the use of renewable resources (such as bio-hydrogen production) will be a novel and promising method. In addition, the production of biohydrogen is likely to have a positive impact on the global energy market, thereby using cheap renewable carbon sources to produce energy. In cluster 2, the frequently-occurring keyword “dark fermentation” indicates that among various hydrogen production methods (including direct biophotolysis, indirect biophotolysis, photo-fermentation, and dark fermentation), dark fermentation is considered the most efficient method because it No external energy is required, and the hydrogen production rate is faster than other methods [21]. Combining all the keywords related to biohydrogen, the discovery of biohydrogen production undoubtedly played an important role in the current FW field, and it attracted widespread attention from 2000 to 2010. Cluster (C1): Compared with other clusters, the main key hot spots of cluster 1 appear more “younger”, mainly after 2010. The main keywords included are “China” (1997), “system” (2002), “management” (2003), “greenhouse gas emissions” (2012), “environmental impact” (2013), and “life-cycle assessment” (2012), “sustainability (2013)”. C1′s primary task is sustainable development, which coincides with China’s development philosophy, and China has also made outstanding contributions to this research direction. C1 was proposed earlier (1998), but it has gradually become a frontier hot spot since 2010. The research period of the entire field is relatively large, and new research hot spots continue to emerge after 2010. It can be seen from the articles included in C1 that to achieve sustainable development, first determine the impact of FW on the environment and improve the evaluation model system. Since LCA will consider the direct and indirect impacts of the food supply chain “from the cradle to the grave” and add them to the local and global impacts, LCA is an important choice for a comprehensive environmental sustainability assessment [9]. Research shows that FW will directly or indirectly emit many greenhouse gases, accounting for 6.8% of greenhouse gas emissions, which aggravates global climate problems [42].Currently, LCA has not been fully developed, and there are obvious shortcomings in modeling. For example, the subjectivity of the model boundary, uncertainty, and certain assumptions to get meaningful results [9].In addition to these, establishing a more stable and targeted FW decision support model is still the focus of future research. Second, we should pay attention to the difference of FW and propose corresponding strategies for changing the environment. Due to differences in economy and production capacity, FW in developing countries mainly occurs on the production and supply sides. In contrast, 50% of FW in developed countries occurs on the consumer side at the household level [24]. It is very effective to pay more attention to the behavior of consumers and effectively intervene at the corresponding stage [24]. Such as adjusting the size of the plate (reducing the FW by 57%) [43], increasing the types of vegetable diets in schools (reducing the FW by 28%) [18]. As for the government, it is necessary to improve the level of food quality supervision and clarify legal obligations. For example, the correct classification of waste) and economic incentives (charges only for mixed residual waste, which are completely partly free) [24]; these have good effects on reducing FW production. “Sustainable development” clearly pointed out that the sustainable management of FW has become a focus of FW research and has attracted the attention of the entire international community: the United Nations Conference on Sustainable Development (Rio de Janeiro) announced the calculation of reducing food waste by half in 2030. This is also part of the global “zero hunger challenge” [2].

## 5. Conclusions

- FW research progress can be roughly divided into four stages: primary development stage (before 2007), consolidation and stability stage (2008–2014), and rapid development stage (2015 to present).
- FW research results are unevenly distributed worldwide, and mainly concentrated in Europe, America, and East Asian countries. There are few research results from Africa. China has become the country with the highest article productivity. In addition, research institutions in South Korea, the United States, and France are all leading in CPP, which shows that the average quality of their papers is high. The hot research area of FW has changed from European and American countries to East Asian countries.
- There are 8298 research articles in 8064 journals in a total of 176 WoS subject categories. Research in the FW field is mainly concentrated in the fields of environment, energy, biotechnology, and sustainability. All publications are concentrated in some journals, such as *Bioresource Technology*, *Waste Management*, and *Journal of Cleaner Production*.
- By systematically analyzing the distribution of the authors’ keywords, it can be concluded that the research in the FW field is mainly divided into five areas, namely “anaerobic digestion or biogas”, “biological hydrogen production”, and “aerobic composting”. “system management” and “generation of value-added products”. Combined with the research on the timeline of keywords, it can be seen that the processing technology of FW, the “anaerobic digestion” composting” and the refined control of “biological hydrogen production” has attracted wide attention from researchers from various countries before 2010. After this, in 2010, management methods aimed at “sustainable development” became the focus of research.
- In the future, research on food waste from the food production side may provide new possibilities for “anti-food waste”. The whole supply–demand chain management methods, including “supply chain tracking network” and “life cycle assessment“, would become research hotspots. Efficient and multitask biological value-added transformation technologies, systematic decision support models, and different management strategies for different groups may become the forefront of research. Meanwhile, the mechanism research is still not neglected by researchers, and the whole process control of food “production–classification–collection–disposal” by managers is still an essential guarantee for the large-scale promotion of food waste resources.

## Figures and Tables

**Table 1:** Top 10 organizations with the most publications in food waste studies.

Rank	Organization	Country	Ps ^①^	P ^②^	H ^③^	CPP ^④^
1	Chinese Academy of sciences	China	174	2.158	30	19.17
2	Tsinghua University	China	162	2.009	35	27.9
3	Tongji University	China	121	1.500	32	25.35
4	Council of Scientific Industrial Research	India	102	1.265	28	20.11
5	China Agricultural University	China	93	1.153	27	24.08
6	INRAE	France	93	1.153	31	34.14
7	University of California System	USA	90	1.116	27	35.16
8	KAIST	Korea	81	1.004	30	41.42
9	University of Science Technology Beijing	China	79	0.980	20	17.67
10	Indian Institute of Technology System	India	77	0.955	21	23.86

Notes: ① publications; ② proportion; ③ h-index; ④ citation per paper. INRAE means French National Institute of Agriculture, Food and Environment. KAIST denotes Korea Institute of Science and Technology.

## Data Availability

No new data were created or analyzed in this study. Data sharing is not applicable to this article.

## Appendix A

**Table A1:** Types of literature in FW studies.

Rank	Type of Literature	Frequency	Proportion (%)
1	Article	6784	81.755
2	Proceedings paper	1050	12.654
3	Review	626	7.544
4	Meeting abstract	103	1.241
5	Editorial material	56	0.675
6	Early access	37	0.446
7	News item	35	0.422
8	Book review	16	0.193
9	Correction	15	0.181
10	Letter	8	0.096
11	Book chapter	6	0.072
12	Data paper	3	0.036
Total		8298	

**Table A2:** Top 10 authors with the most publications in FW studies.

Rank	Author	Country	Organization	TP ^①^	P ^②^	H ^③^	CPP ^④^	TLS ^⑤^
1	Wang Qunhui	China	University of Science and Technology Beijing	51	0.75	28	15.18	153
2	Kim Donghoon	South Korea	Inha University	38	0.56	28	27.79	111
3	Lin, Carol Sze Ki	China	City University of Hong Kong	38	0.56	33	47.89	70
4	Wong Jonathan C.W.	China	Hong Kong Baptist University	38	0.56	25	35.84	61
5	Li Yuyou	Japan	Tohoku University	37	0.55	26	34.00	91
6	Mohan S. Venkata	India	Indian Institute of Chemical Technology	33	0.49	10	33.00	10
7	Ok Yong Sik	South Korea	Kangwon National University	32	0.47	72	36.44	150
8	Tsang Daniel C.W.	China	Hong Kong Polytechnic University	31	0.46	4	38.35	149
9	Zhang Ruihong	China	Harbin Institute of Technology	29	0.43	39	62.55	89
10	Ma Hongzhi	China	University of Science and Technology Beijing	28	0.41	23	11.89	78

Notes: ① total publications; ② percentage, %; ③ h-index; ④ citations per paper; ⑤ total link strength.

**Table A3:** Top 10 journals with the most publications in FW studies.

Rank	Journal Title	Country	Ps ^①^	P ^②^	H ^③^	TC ^④^	CPP ^⑤^	CI ^⑥^
1	*Bioresource Technology*	Netherlands	797	9.883	77	27,876	34.79	SCIE
2	*Waste Management*	United Kingdom	500	6.2	65	15,971	31.94	SCIE
3	*Journal of Cleaner Production*	Netherlands	294	3.646	40	7401	25.17	SCIE
4	*International Journal of Hydrogen Energy*	United Kingdom	238	2.951	48	10,181	42.78	SCIE
5	*Sustainability*	Switzerland	149	1.848	18	1395	9.36	SCIE/SSCI
6	*Journal of Environmental Management*	United States	137	1.699	29	3319	24.23	SCIE
7	*Resources Conservation and Recycling*	Netherlands	131	1.625	33	3593	27.43	SCIE
8	*Waste Management Research*	United Kingdom	112	1.389	24	1905	17.01	SCIE
9	*Science of the Total Environment*	Netherlands	99	1.228	20	2881	29.1	SCIE
10	*Water Science and Technology*	United Kingdom	92	1.141	23	1648	17.91	SCIE

Notes: ① publications; ② percentage, %; ③ h-index; ④ total citations; ⑤ citations per paper; ⑥ citation index.

**Table A4:** Top 10 disciplines with most publications in FW studies.

Rank	Discipline	Ps ^①^	H ^②^	CPP ^③^
1	Environmental sciences	2725	96	21.14
2	Energy fuels	1924	103	20.44
3	Engineering environmental	1733	89	23.53
4	Biotechnology applied microbiology	1438	87	29.37
5	Green sustainable science technology	898	65	22.65
6	Agricultural engineering	895	79	33.1
7	Engineering chemical	701	53	19.7
8	Food science technology	568	51	20.41
9	Chemistry physical	302	53	39.69
10	Chemistry multidisciplinary	281	33	16.56

Notes: ① publications; ② h-index; ③ citations per paper.
